# Essential Updates 2024–2026: Advances in Colorectal Cancer Surgery

**DOI:** 10.1002/ags3.70260

**Published:** 2026-08-01

**Authors:** Hiroyasu Kagawa, Yusuke Kinugasa

**Affiliations:** ^1^ Department of Gastrointestinal Surgery Institute of Science Tokyo Tokyo Japan

**Keywords:** colorectal cancer, intracorporeal anastomosis, rectal cancer, robotic surgery, transanal total mesorectal excision

## Abstract

Surgical innovation in colorectal cancer is increasingly judged not by technical feasibility alone, but by whether it improves oncological outcomes, preserves function, reduces morbidity, or makes difficult procedures more reproducible. This structured narrative review examines major surgical studies published from January 2024 through July 2026, while retaining earlier landmark reports when needed to interpret safety and implementation. The strongest recent randomized evidence concerns robot‐assisted rectal cancer surgery. In the REAL trial, robotic surgery improved 3‐year locoregional control, disease‐free survival, and early functional recovery compared with laparoscopy in middle and low rectal cancer, although overall survival did not differ and generalizability beyond experienced Chinese centers remains uncertain. For transanal total mesorectal excision, noninferiority in the TaLaR trial must be interpreted in light of its −10% margin, the experience of participating centers, and early reports of multifocal local recurrence during implementation. Individual trials of indocyanine green fluorescence angiography have yielded mixed results; however, a 2026 meta‐analysis of nine randomized trials found lower leakage rates overall and after left‐sided and rectal resections, but not after right‐sided colectomy, supporting procedure‐specific use. Robotic colectomy is feasible but has not clearly surpassed mature laparoscopy. Intracorporeal anastomosis is associated with faster recovery and greater flexibility in extraction‐site selection and may reduce extraction‐site hernia, whereas long‐term oncological equivalence has not yet been established. Across these fields, benefit appears context‐specific and closely linked to case selection, surgeon experience, technical standardization, and structured audit.

## 1 Introduction

Minimally invasive surgery has become established in colorectal cancer surgery, shifting the central question from technical feasibility to whether a specific approach improves oncological outcomes, preserves function, reduces morbidity, or makes difficult procedures more reproducible. Because laparoscopic and robot‐assisted surgery require advanced and costly technology, their value should be judged against mature alternatives rather than technical novelty alone.

This structured narrative review examines studies published from 2024 through July 2026 on robot‐assisted rectal and colon surgery, technically demanding procedures (lateral lymph node dissection, pelvic exenteration), transanal total mesorectal excision (TaTME), indocyanine green (ICG) fluorescence angiography, low rectal reconstruction, and intracorporeal anastomosis (ICA). Earlier landmark studies are included when needed to interpret safety or implementation. We focus on evidence likely to influence operative decision‐making, patient selection, and responsible adoption. Key studies are summarized by topic in Tables 1, 2, 3.

**TABLE 1:** Key studies of robot‐assisted rectal cancer surgery.

Study	Year/design	Sample	Comparison/setting	Main finding	Major limitation
ROLARR [1]	2017; multicenter RCT	471	Robotic vs. laparoscopic rectal resection	No significant reduction in conversion to open surgery	Early adoption phase; variable robotic experience
REAL [2]	2025; multicenter superiority RCT	1171	Robotic vs. laparoscopic surgery for middle/low rectal cancer	Lower 3‐year locoregional recurrence and higher DFS; better early functional recovery; no OS difference	Single country; experienced surgeons; cost‐effectiveness uncertain
VITRUVIANO [3]	2024; multicenter phase II	191	Robot‐assisted surgery for advanced rectal cancer	No conversion; CRM positivity 4.6%; grade III–IV complications 6.3%	Single‐arm study without laparoscopic comparator
Hanaoka et al. [4]	2025; nationwide real‐world cohort	28 711	Open, laparoscopic, and robotic rectal cancer surgery	Robotic surgery associated with favorable adjusted survival	Residual confounding, treatment‐era effects, and institutional selection
LANDMARC [5]	2026; prospective multicenter study	399	Laparoscopic, robotic, and transanal approaches	After matching, ejaculatory dysfunction was lower after robotic than laparoscopic surgery	Nonrandomized approach selection; male patients only
ROSEMARY [6]	2025; multicenter retrospective cohort	1210	Robotic ultralow anterior resection or intersphincteric resection	Very low conversion and acceptable major morbidity in technically demanding low rectal surgery	No comparator; high‐volume centers
Yatabe et al. [7]	2024; multicenter propensity‐matched analysis	261	Robotic vs. laparoscopic vs. open pelvic exenteration	Robotic exenteration feasible in selected patients	Highly selected cases; heterogeneous pelvic malignancies

Abbreviations: CRM, circumferential resection margin; DFS, disease‐free survival; OS, overall survival; RCT, randomized controlled trial.

**TABLE 2:** Randomized trials of ICG fluorescence angiography for anastomotic perfusion assessment.

Study	Year/design	Population	Sample	Anastomotic leakage	Interpretation/limitation
EssentiAL [8]	2023; phase III RCT	Minimally invasive rectal cancer surgery	839	7.6% with ICGFA vs. 11.8% with standard assessment	Supported benefit in rectal anastomoses; subjective fluorescence interpretation
AVOID [9]	2024; multicenter phase III RCT	Mixed minimally invasive colorectal procedures	982 enrolled; 931 ITT	7% vs. 9%; RR 0.77 (95% CI 0.50–1.20)	No significant overall benefit; mixed operative sites and indications
IntAct [10]	2025; multicenter RCT	Rectal resection	766 randomized; 698 analyzed	10.5% vs. 15.2%; adjusted OR 0.67 (95% CI 0.42–1.06)	Numerical reduction but adjusted analysis not significant; heterogeneous techniques
ICG‐COLORAL [11]	2025; multicenter RCT	Colorectal resection excluding low anterior resection	1136	Overall 5.8% vs. 7.9%; left‐sided 5.2% vs. 9.5%	No significant overall benefit; left‐sided subgroup suggested greater benefit

*Note:* A subsequent meta‐analysis of nine randomized trials found significant reductions in leakage overall and after left‐sided and rectal resections, but not after right‐sided resection [12].

Abbreviations: CI, confidence interval; ICGFA, indocyanine green fluorescence angiography; ITT, intention‐to‐treat; OR, odds ratio; RCT, randomized controlled trial; RR, risk ratio.

**TABLE 3:** Key studies of intracorporeal versus extracorporeal anastomosis.

Study	Year/design	Sample	Comparison	Main finding	Major limitation/status
ICAN [13]	2025; nationwide multicenter cohort	1245	ICA vs. extracorporeal anastomosis in laparoscopic colectomy	Less blood loss and vascular injury, faster bowel recovery, and shorter stay with ICA; longer operating time	Observational design; institutional experience and case selection
Wei et al. [14]	2024; meta‐analysis of 7 RCTs	720	ICA vs. extracorporeal anastomosis in laparoscopic right hemicolectomy	Faster gastrointestinal recovery and fewer wound‐related complications; no clear reduction in leakage	Heterogeneity in techniques and outcome definitions
Hemi‐D‐TREND [15]	2026; prospective multicenter cohort	438	ICA vs. extracorporeal anastomosis after laparoscopic right colectomy	Leakage below 2% in both groups; lower conversion and shorter stay with ICA	Nonrandomized; approach varied by center
ANCOR [16]	2026; prospective multicenter nonrandomized trial	300	ICA vs. extracorporeal anastomosis in robotic/laparoscopic right colectomy	Extraction‐site hernia at 2 years 1.9% vs. 10.1%	Included benign and malignant disease; extraction site confounded with ICA
INEX [17]	2025; multicenter RCT protocol	Planned 1400	ICA vs. extracorporeal anastomosis in stage II/III colon cancer	Designed to evaluate long‐term oncological outcomes	Results pending
COLOR IV [18]	2025; multicenter RCT protocol	Planned 1158	ICA vs. extracorporeal ileocolic anastomosis after laparoscopic right colectomy	Will assess 30‐day leakage and 3‐year DFS under quality assurance	Results pending

Abbreviations: DFS, disease‐free survival; ICA, intracorporeal anastomosis; RCT, randomized controlled trial.

## 2 Methods for Literature Selection

This structured narrative review evaluated studies of colorectal cancer surgery published in English from January 2024 through July 2026. PubMed/MEDLINE searches combined the terms “colorectal cancer,” “rectal cancer,” “colon cancer,” “robotic surgery,” “total mesorectal excision,” “lateral lymph node dissection,” “pelvic exenteration,” “TaTME,” “indocyanine green fluorescence angiography,” and “intracorporeal anastomosis.” Randomized trials, prospective multicenter studies, nationwide or registry‐based analyses, and large comparative cohorts were prioritized. Earlier studies were retained when they were needed to explain the development of a technique or a recognized safety concern. Because this was a narrative review, no formal risk‐of‐bias assessment or quantitative synthesis was undertaken.

## 3 Advances in Rectal Cancer Surgery

### 3.1 Outcomes of Robotic Rectal Surgery

Early randomized evidence did not demonstrate a clear clinical advantage of robot‐assisted rectal cancer surgery. In the ROLARR trial, robot‐assisted surgery did not significantly reduce conversion to open surgery compared with conventional laparoscopy [1]. This finding was influential because conversion had been regarded as the most direct measure of whether articulated instruments, tremor filtration, and a stable three‐dimensional view could overcome the limitations of straight laparoscopic instruments in the pelvis. However, ROLARR was conducted during an early phase of robotic adoption, and the participating surgeons had varying levels of robotic experience. Moreover, the trial was not designed to evaluate superiority in long‐term oncological or functional outcomes.

The REAL trial substantially advanced the evidence base. Conducted at 11 centers in China, the trial included patients with nonmetastatic middle or low rectal adenocarcinoma classified as cT1–3, N0–1, or ycT1–3, Nx, and 1171 patients were included in the primary analysis [2]. Compared with laparoscopic surgery, robotic surgery was associated with a significantly lower 3‐year locoregional recurrence rate (1.6% vs. 4.0%; hazard ratio [HR], 0.45; 95% confidence interval [CI], 0.22–0.92; *p* = 0.03) and a significantly higher 3‐year disease‐free survival rate (87.2% vs. 83.4%; HR, 0.74; 95% CI, 0.56–0.98; *p* = 0.04). Three‐year overall survival did not differ significantly (94.7% vs. 93.0%). Urinary function, sexual function in both men and women, and bowel function were more favorable in the robotic group at 3 and 6 months after surgery; urinary function and male sexual function remained better at 12 months. These findings may reflect more precise pelvic dissection and improved preservation of anatomical planes. Nevertheless, the trial involved experienced surgeons within a single healthcare system, and whether comparable results can be reproduced in institutions with different case volumes, training systems, and case complexity remains uncertain.

Prospective and real‐world studies from Japan provide complementary evidence. In the multicenter phase II VITRUVIANO study of advanced rectal cancer, no conversions to open surgery were reported, the circumferential resection margin positivity rate was 4.6%, and the rate of grade III‐IV complications was 6.3% [3]. A large real‐world study using the Medical Data Vision database compared open, laparoscopic, and robot‐assisted surgery in 28 711 patients with clinical stage I–III rectal cancer. The 5‐year overall survival rates were 95% after robotic surgery, 89% after laparoscopic surgery, and 81% after open surgery, while the corresponding relapse‐free survival rates were 93%, 86%, and 77%, respectively [4]. With robotic surgery as the reference, multivariable analysis associated laparoscopic and open surgery with higher mortality risks (HR, 2.18; 95% CI, 1.69–2.81 and HR, 3.96; 95% CI, 3.03–5.19, respectively). A separate real‐world cohort restricted to locally advanced rectal cancer similarly reported favorable 5‐year survival after robot‐assisted resection [19]. Although clinically encouraging, these large differences should be interpreted cautiously because retrospective comparisons cannot fully account for treatment period, patient selection, surgeon expertise, and institutional characteristics. Analyses using the Diagnosis Procedure Combination database also compared short‐term outcomes between robotic and laparoscopic surgery and highlighted a potential trade‐off between perioperative outcomes and the use of healthcare resources and costs [20, 21]. These observational associations are compatible with the direction of the REAL trial findings but should not be regarded as confirmatory because of residual confounding, treatment‐era effects, and differences in institutional expertise.

Functional outcomes are also central to assessing the value of robotic surgery. The prospective multicenter LANDMARC study evaluated 399 men and reported overall incidences of erectile dysfunction and ejaculatory dysfunction at 12 months after surgery of 34.7% and 29.8%, respectively [5]. After propensity score matching, ejaculatory dysfunction occurred significantly less often after robotic surgery than after laparoscopic surgery (25.0% vs. 40.9%). These findings suggest that robotic surgery may contribute to preservation of at least some aspects of male sexual function by facilitating more precise dissection around the pelvic autonomic nerves.

The REAL trial provides randomized evidence that robotic surgery can improve 3‐year locoregional control, disease‐free survival, and early functional recovery in patients with middle and low rectal cancer when performed by experienced surgeons. Confirmation in other healthcare systems and evaluation of cost‐effectiveness remain necessary before general superiority over laparoscopy can be concluded.

### 3.2 Robotic Surgery for Technically Demanding Rectal Cancer

The clinical rationale for robot‐assisted surgery is strongest when oncological dissection must be maintained within a narrow, deep, or otherwise unforgiving operative field. The multicenter ROSEMARY study evaluated robot‐assisted ultralow anterior resection and intersphincteric resection for low rectal cancer at 31 high‐volume institutions in Japan [6]. Robot‐assisted ultralow rectal surgery was performed safely, with a very low conversion rate and acceptable rates of major morbidity and mortality. These results support the feasibility of robot‐assisted surgery for procedures in which distal transection, intersphincteric dissection, and reconstruction must be performed in the deepest part of the pelvis. The study did not establish superiority over other approaches, but it identified a clinical setting in which articulated instruments and stable exposure may offer greater practical value than in a straightforward TME.

The method of reconstruction is also important in very low rectal surgery. A subanalysis of the ULTIMATE study compared hand‐sewn and stapled anastomoses in 135 patients with cT1–2, N0, M0 tumors located 4–5 cm from the anal verge [22]. Although postoperative complications, oncological outcomes, and urinary and sexual function were comparable between the groups, stapled anastomosis was associated with consistently better anorectal function during the 3‐year follow‐up. These findings do not imply that stapled anastomosis is invariably preferable. The distal resection margin, sphincter involvement, luminal diameter, and technical feasibility still determine which reconstruction can be performed safely. They do show, however, that even after an oncologically adequate resection, the choice of anastomotic technique may have a lasting effect on bowel function and quality of life.

Lateral lymph node dissection (LLND) is another procedure in which relatively small technical differences may have important oncological and functional consequences. Dissection proceeds along the internal iliac and obturator compartments, placing the ureter, obturator nerve, pelvic autonomic nerves, and major vessels at risk. A Japanese multicenter retrospective study supported a role for LLND in local control in selected patients [23], although the nonrandomized design and treatment heterogeneity limit causal interpretation. A recent report has also provided a more standardized anatomical description of the procedure [24]. The robotic platform may improve exposure and facilitate fine dissection around the internal iliac branches and pelvic plexus, but these advantages remain dependent on a consistent operative strategy and a detailed understanding of lateral pelvic anatomy. A study of urinary function after robot‐assisted rectal cancer surgery found that both autonomic nerve resection and LLND were independently associated with postoperative urinary dysfunction [25]. Although the robotic platform may facilitate precise nerve‐preserving dissection when oncologically appropriate, nerve resection inevitably carries a risk of urinary dysfunction. LLND should therefore be performed with careful attention to the balance between oncological clearance and functional preservation.

Pelvic exenteration is one of the most technically demanding procedures in colorectal surgery. A Japanese multicenter propensity score‐matched analysis compared robotic, laparoscopic, and open pelvic exenteration for locally advanced or recurrent pelvic malignancies [7]. The findings suggest that a minimally invasive robotic approach is feasible in carefully selected patients. Nevertheless, achieving a safe and complete resection must remain the primary objective. Multidisciplinary planning, adequate preparation for vascular or urinary reconstruction, and timely conversion to open surgery when necessary are more important than maintaining a minimally invasive approach. Robotics may broaden the surgical options available to experienced teams, but it should not be used to extend the indications for surgery beyond the limits of sound oncological judgment.

Future studies of robot‐assisted rectal cancer surgery should therefore avoid treating all rectal resections as a single category and instead address questions specific to the complexity of the procedure and the patient. Cases involving low tumors, a narrow male pelvis, obesity, lateral lymph node dissection, intersphincteric resection, or multivisceral resection should be evaluated separately from standard TME. The true value—and the limitations—of the robotic platform are likely to be most apparent in these technically demanding settings.

### 3.3 TaTME: Oncological Promise, Implementation Risk, and Current Role

TaTME was developed to improve distal access in low rectal cancer, but its assessment has shifted from feasibility to oncological safety, the learning curve, and the conditions required for responsible implementation.

In the TaLaR randomized trial, 3‐year disease‐free survival was 82.1% after TaTME and 79.4% after laparoscopic TME, corresponding to an absolute between‐group difference of 2.7 percentage points (97.5% CI, −3.0 to 8.1 percentage points), with the lower confidence limit exceeding the prespecified noninferiority margin of −10 percentage points [26]. The relatively wide margin and conduct within experienced centers should be considered when applying these results more broadly. A 2025 meta‐analysis also suggested lower conversion rates and favorable circumferential resection margin outcomes, although study heterogeneity and uncertainty regarding functional outcomes remained [27].

Early implementation raised serious concerns about local control. Norway suspended TaTME after local recurrence occurred in 12 of 157 patients (7.6%), often with multifocal pelvic disease [28]. In the Netherlands, the recurrence rate among the first ten cases at each center was 10.0%, with multifocal recurrence in eight of 12 patients [29]. A subsequent audit found that local recurrence decreased from 12.5% during the early learning phase to 3.4% with greater experience [30]. Incomplete purse‐string closure, tumor‐cell spillage, incorrect dissection planes, and excessive tumor manipulation have been proposed as possible mechanisms. A Japanese nationwide cohort reported a 3‐year cumulative local recurrence rate of 6.8% and identified factors associated with recurrence [31].

TaTME should therefore be reserved for low rectal tumors in which distal access is genuinely difficult and introduced only through structured training, supervised cases, formal proctorship, and continuing audit. It should address a defined anatomical problem rather than serve as a routine alternative to laparoscopic or robotic TME.

## 4 Anastomotic Perfusion Assessment

Adequate perfusion is essential for anastomotic healing after rectal resection, although tension, venous drainage, anastomotic level and technique, and the condition of the distal stump are also important. ICG fluorescence angiography provides real‐time perfusion information that cannot be obtained reliably from bowel color or arterial pulsation alone.

Four major randomized trials have produced differing results (Table 2). The EssentiAL trial demonstrated a reduction in leakage after rectal cancer surgery [8], whereas AVOID found no significant overall benefit across mixed colorectal procedures [9]. IntAct showed a numerical reduction after rectal resection that did not reach significance in adjusted analysis [10], and ICG‐COLORAL found no significant overall benefit after excluding low anterior resection but suggested a larger effect in left‐sided surgery [11].

A 2026 meta‐analysis of nine randomized trials involving 4754 patients found lower leakage rates overall and after left‐sided, rectal, and low anterior resections (RR, 0.62–0.66), with no clear benefit after right‐sided resection [12]. Current evidence therefore supports procedure‐specific use, particularly for left‐sided and rectal anastomoses, rather than uniform application to all colorectal operations. Standardization of ICG dose, timing, image interpretation, and quantitative thresholds remains necessary.

## 5 Advances in Colon Cancer Surgery

### 5.1 Robotic Colectomy

Because most colonic procedures can already be performed effectively using established laparoscopic techniques, the rationale for robotic surgery in colon cancer is less compelling than it is in rectal cancer. Most available evidence has focused on right colectomy, where the robotic platform may facilitate central vascular dissection, complete mesocolic excision, multiquadrant mobilization, and intracorporeal reconstruction. These are meaningful technical advantages, but the appropriate comparator remains a mature laparoscopic approach with low conversion and complication rates.

A Japanese multicenter prospective cohort demonstrated the safety and feasibility of robot‐assisted right colectomy [32]. The prospective phase II ROBOCOLO trial similarly supported robotic surgery as a practical option for resectable right‐sided colon cancer [33]. Neither study established superiority over laparoscopy, but both showed that a standardized robotic approach can achieve acceptable short‐term and pathological outcomes.

Short‐term outcomes with the hinotori Surgical Robot System have also been reported [34]. The introduction of additional robotic platforms may reduce dependence on a single system and promote competition in instrumentation and cost. At the same time, each new platform introduces its own learning curve. Platform‐specific performance should therefore be evaluated separately from the quality of the colectomy itself.

At present, the evidence supports the feasibility of robotic colectomy rather than its routine use as a replacement for laparoscopy. Its potential benefit may be greater in patients with complex vascular anatomy, obesity, a need for extended lymphadenectomy, or a planned intracorporeal anastomosis. Future comparative studies should focus on these subgroups and should include total cost, surgeon workload, and long‐term outcomes.

### 5.2 Intracorporeal Versus Extracorporeal Anastomosis

ICA avoids exteriorization of the bowel before reconstruction, allowing mobilization to be determined by the resection rather than the extraction site. The specimen can be removed through an umbilical or Pfannenstiel incision, potentially reducing mesenteric traction and facilitating avoidance of a midline incision. The trade‐off is greater technical complexity and the need for secure stapling or suturing with careful contamination control.

Recent evidence is summarized in Table 3. In the nationwide ICAN cohort, ICA required longer operating time but was associated with less blood loss, fewer adjusted intraoperative vascular injuries, earlier bowel recovery, and shorter hospitalization [13]. A meta‐analysis of randomized trials similarly found faster gastrointestinal recovery and fewer wound‐related complications without a clear reduction in leakage [14]. In Hemi‐D‐TREND, leakage was below 2% with both techniques; ICA was associated with lower conversion and shorter stay, but not fewer severe complications [15]. ANCOR reported fewer extraction‐site hernias after ICA, although the nonrandomized design and close relationship between ICA and non‐midline extraction limit causal attribution [16].

Long‐term oncological equivalence has not yet been established. The INEX trial is evaluating long‐term outcomes in stage II/III colon cancer [17], and COLOR IV will assess both 30‐day leakage and 3‐year disease‐free survival after laparoscopic right colectomy within a formal quality‐assurance program [18].

Robotic articulation may facilitate bowel orientation and closure of the common enterotomy, but laparoscopic ICA is already reproducible in experienced hands. Outcomes after robotic colectomy with ICA reflect several linked choices—the access platform, vascular dissection, reconstruction, and extraction site—and should not be attributed to the robotic system alone. The clearest current benefits of ICA appear to concern recovery and extraction‐site morbidity rather than anastomotic security.

## 6 Future Directions

Several themes emerge. First, benefit is usually concentrated in a defined clinical setting. Robotics appears most relevant in the deep pelvis and other technically demanding procedures; ICG fluorescence angiography is supported most clearly for left‐sided and rectal anastomoses; and the principal advantages of ICA may be faster recovery and flexibility in extraction‐site selection. Broad comparisons across all operations can obscure these subgroups.

Second, implementation is part of the intervention. The TaTME experience showed that technical completion does not guarantee acceptable oncological outcomes during early dissemination. Outcomes with robotics and ICA also depend on case selection, team coordination, standardization, and experience. Trials and registries should report surgeon volume, credentialing, and learning‐phase outcomes explicitly.

Third, technical measures such as operating time, conversion, and blood loss should be assessed alongside cost, urinary, sexual and bowel function, incisional hernia, quality of life, and long‐term cancer control. Studies should also separate the effect of a device from the wider surgical strategy: fluorescence is useful only when it changes management, and the apparent benefit of ICA may partly reflect the choice of extraction incision.

This review has limitations. It is a structured narrative rather than a systematic review, relied primarily on PubMed/MEDLINE, and did not include formal risk‐of‐bias assessment or de novo quantitative synthesis. Study selection may therefore reflect author judgment, and relevant evidence outside the predefined topics may have been omitted.

## 7 Conclusions

Recent evidence has moved evaluation of colorectal surgical innovation beyond feasibility toward oncological, functional, and patient‐centered outcomes. The REAL trial supports improved 3‐year locoregional control, disease‐free survival, and early functional recovery with robotic surgery for middle and low rectal cancer in experienced centers, but broader confirmation and cost‐effectiveness data are required. Robotics may be particularly useful in technically demanding pelvic surgery, whereas TaTME should remain selective and subject to structured training and audit. Pooled randomized evidence supports ICG fluorescence angiography for left‐sided and rectal anastomoses but not routine use in all colorectal procedures. Robotic colectomy remains feasible without proven general superiority over laparoscopy. ICA offers advantages in recovery and extraction‐site selection, while long‐term oncological equivalence has not yet been established. Future studies should identify the patients most likely to benefit and report learning‐curve, functional, economic, and long‐term oncological outcomes.

## Author Contributions

H.K.: Conceptualization; Investigation; Writing – original draft. Y.K.: Supervision; Writing – review and editing.

## Funding

The authors have nothing to report.

## Ethics Statement

As this article is a narrative review of previously published studies, ethics committee approval and informed consent were not required.

## Conflicts of Interest

Y.K. is an editorial board member of the Annals of Gastroenterological Surgery. Y.K. received speaker honoraria from Intuitive Surgical Inc., Johnson & Johnson K.K., and Medtronic Japan. H.K. received speaker honoraria from Intuitive Surgical Inc. and Johnson & Johnson K.K.

## Data Availability

Data sharing not applicable to this article as no datasets were generated or analysed during the current study.

## References

[1] D. Jayne , A. Pigazzi , H. Marshall , et al., “Effect of Robotic‐Assisted vs Conventional Laparoscopic Surgery on Risk of Conversion to Open Laparotomy Among Patients Undergoing Resection for Rectal Cancer: The ROLARR Randomized Clinical Trial,” Journal of the American Medical Association 318, no. 16 (2017): 1569–1580. 10.1001/jama.2017.7219 PMC5818805 29067426

[2] Q. Feng , W. Yuan , T. Li , et al., “Robotic vs Laparoscopic Surgery for Middle and Low Rectal Cancer: The REAL Randomized Clinical Trial,” Journal of the American Medical Association 334, no. 2 (2025): 136–148. 10.1001/jama.2025.8123 PMC12131176 40455621

[3] A. Hamabe , I. Takemasa , M. Kotake , et al., “Feasibility of Robotic‐Assisted Surgery in Advanced Rectal Cancer: A Multicentre Prospective Phase II Study (VITRUVIANO Trial),” BJS Open 8, no. 3 (2024): zrae048. 10.1093/bjsopen/zrae048 PMC11195309 38913419

[4] M. Hanaoka , H. Kagawa , A. Igarashi , et al., “Short‐ and Long‐Term Outcomes of Open, Laparoscopic, and Robot‐Assisted Surgery for Rectal Cancer,” Annals of Gastroenterological Surgery 9, no. 5 (2025): 1017–1028. 10.1002/ags3.70024 PMC12414592 40922920

[5] M. Numata , T. Yamaguchi , A. Shiomi , et al., “Prospective Multicenter Comprehensive Survey on Male Sexual Dysfunction Following Laparoscopic, Robotic, and Transanal Approaches for Rectal Cancer (The LANDMARC Study),” Annals of Surgery 283, no. 5 (2026): 869–877. 10.1097/SLA.0000000000006574 PMC13056404 39435538

[6] M. Miyo , A. Shiomi , S. W. Lee , et al., “Short‐Term Outcomes of Robot‐Assisted Ultralow Anterior Resection and Robot‐Assisted Rectectomy With Transanal Anastomosis for Rectal Cancer in Japan: A Multicenter Retrospective Cohort Study (ROSEMARY Study),” Surgery 184 (2025): 109414. 10.1016/j.surg.2025.109414 40482436

[7] Y. Yatabe , M. Hanaoka , R. Hanazawa , et al., “Robotic Versus Open and Laparoscopic Pelvic Exenterations for Pelvic Cancer: A Multicenter Propensity‐Matched Analysis in Japan,” Surgical Endoscopy 38, no. 8 (2024): 4390–4401. 10.1007/s00464-024-10966-w 38886231

[8] J. Watanabe , I. Takemasa , M. Kotake , et al., “Blood Perfusion Assessment by Indocyanine Green Fluorescence Imaging for Minimally Invasive Rectal Cancer Surgery (EssentiAL Trial): A Randomized Clinical Trial,” Annals of Surgery 278, no. 4 (2023): e688–e694. 10.1097/SLA.0000000000005907 PMC10481925 37218517

[9] R. A. Faber , R. P. J. Meijer , D. H. M. Droogh , et al., “Indocyanine Green Near‐Infrared Fluorescence Bowel Perfusion Assessment to Prevent Anastomotic Leakage in Minimally Invasive Colorectal Surgery (AVOID): A Multicentre, Randomised, Controlled, Phase 3 Trial,” Lancet Gastroenterology & Hepatology 9, no. 10 (2024): 924–934. 10.1016/S2468-1253(24)00198-5 39151436

[10] D. Jayne , J. Croft , N. Corrigan , et al., “Intraoperative Fluorescence Angiography With Indocyanine Green to Prevent Anastomotic Leak in Rectal Cancer Surgery (IntAct): An Unblinded Randomised Controlled Trial,” Lancet Gastroenterology & Hepatology 10, no. 9 (2025): 806–817. 10.1016/S2468-1253(25)00101-3 40690925

[11] J. K. A. Rinne , H. Huhta , T. Pinta , et al., “Indocyanine Green Fluorescence Imaging in Prevention of Colorectal Anastomotic Leakage: A Randomized Clinical Trial,” JAMA Surgery 160, no. 5 (2025): 486–493. 10.1001/jamasurg.2025.0006 PMC11883591 40042831

[12] E. J. Ryan , O. K. Ryan , N. Corrigan , et al., “Indocyanine Green Fluorescence Angiography for Anastomotic Perfusion Assessment in Colorectal Surgery: A Systematic Review With Meta‐Analysis, Meta‐Regression, and Trial Sequential Analyses,” Lancet Gastroenterology & Hepatology 11, no. 5 (2026): 367–379. 10.1016/S2468-1253(25)00373-5 41871586

[13] T. Yamaguchi , K. Tanaka , J. Watanabe , et al., “Short‐Term Outcomes of Intracorporeal Anastomosis in Laparoscopic Colectomy for Colon Cancer: A Nationwide, Multi‐Institutional Cohort Study in Japan (ICAN Study),” Annals of Gastroenterological Surgery 9, no. 4 (2025): 739–749. 10.1002/ags3.12915 PMC12211091 40607305

[14] P. Wei , Y. Li , J. Gao , et al., “Intracorporeal Versus Extracorporeal Anastomosis in Laparoscopic Right Hemicolectomy: An Updated Systematic Review and Meta‐Analysis of Randomized Control Trials,” Digestive Surgery 41, no. 5–6 (2024): 224–244. 10.1159/000541373 39342943

[15] X. Serra‐Aracil , M. Pascua‐Solé , A. Sánchez , et al., “Intracorporeal vs Extracorporeal Anastomosis in Laparoscopic Right Colectomy for Colon Cancer: A Prospective Multicenter Cohort Study (The Hemi‐D‐TREND Study),” Surgical Endoscopy 40, no. 2 (2026): 1559–1571. 10.1007/s00464-025-12401-0 PMC12881089 41326726

[16] R. K. Cleary , M. Silviera , T. J. Reidy , et al., “Extraction Site Hernia and Short‐Term Outcomes Following Intracorporeal Versus Extracorporeal Anastomosis for Robotic and Laparoscopic Right Colectomy: A Multi‐Center Prospective Trial,” Surgical Endoscopy 40, no. 1 (2026): 502–511. 10.1007/s00464-025-12327-7 PMC12823757 41145697

[17] M. Hanaoka , H. Kagawa , M. Ishiguro , A. Hirakawa , M. Tokunaga , and Y. Kinugasa , “Long‐Term Prognosis of Intracorporeal Versus Extracorporeal Anastomosis in Stage II/III Colorectal Cancer (INEX Study): Study Protocol for a Multicenter Randomized Controlled Trial in Japan,” BMC Cancer 25, no. 1 (2025): 1242. 10.1186/s12885-025-14676-x PMC12309057 40739197

[18] S. Wu , P. Wei , J. Gao , et al., “COLOR IV: A Multicenter Randomized Clinical Trial Comparing Intracorporeal and Extracorporeal Ileocolic Anastomosis After Laparoscopic Right Colectomy for Colon Cancer,” Surgical Endoscopy 39, no. 2 (2025): 1182–1190. 10.1007/s00464-024-11412-7 PMC11794397 39733171

[19] M. Hanaoka , H. Kagawa , A. Igarashi , et al., “Improved 5‐Year Survival With Robot‐Assisted Resection for Locally Advanced Rectal Cancer Compared to Laparoscopic and Open Surgery: A Real‐World Cohort Study,” Colorectal Disease 27, no. 11 (2025): e70278. 10.1111/codi.70278 PMC12568752 41152199

[20] M. Mizoguchi , M. Kizuki , N. Iwata , et al., “Comparison of Short‐Term Outcomes Between Robot‐Assisted and Laparoscopic Rectal Surgery for Rectal Cancer: A Propensity Score‐Matched Analysis Using the Japanese Nationwide Diagnosis Procedure Combination Database,” Annals of Gastroenterological Surgery 7, no. 6 (2023): 955–967. 10.1002/ags3.12707 PMC10623962 37927934

[21] H. Hamamoto , M. Ota , T. Shima , et al., “Comparison of Short‐Term Outcomes and Perioperative Costs in Laparoscopic Versus Robotic Surgery for Rectal Cancers: A Real‐World Cohort Study Using Japanese Nationwide Inpatient Database,” Annals of Gastroenterological Surgery 9, no. 1 (2025): 4–11. 10.1002/ags3.12884 PMC11693608 39759987

[22] M. Numata , J. Watanabe , Y. Tsukada , et al., “Patient‐Reported Outcomes and Surgical Results of Hand‐Sewn Versus Stapled Anastomosis for Lower Rectal Cancer Located 4‐5 Cm From the Anal Verge: A Subanalysis of the Ultimate Study,” Annals of Gastroenterological Surgery 9, no. 6 (2025): 1215–1224. 10.1002/ags3.70063 PMC12586937 41199980

[23] Y. Tanaka , H. Hino , A. Shiomi , et al., “Efficacy of Lateral Lymph Node Dissection for Local Control of Rectal Cancer: A Multicenter Study,” Annals of Gastroenterological Surgery 8, no. 4 (2024): 631–638. 10.1002/ags3.12789 PMC11216789 38957561

[24] M. Uemura , J. Watanabe , A. Shiomi , et al., “Surgical Procedure of Lateral Lymph Node Dissection for Advanced Lower Rectal Cancer,” Annals of Gastroenterological Surgery 9, no. 6 (2025): 1112–1118. 10.1002/ags3.70039 PMC12586956 41199967

[25] S. Arai , H. Kagawa , A. Shiomi , et al., “Effect of Autonomic Nervous System Resection Extent on Urinary Dysfunction in Robotic Rectal Cancer Surgery,” Annals of Gastroenterological Surgery 9, no. 3 (2025): 476–485. 10.1002/ags3.12878 PMC12080188 40385347

[26] Z. Zeng , S. Luo , H. Zhang , et al., “Transanal vs Laparoscopic Total Mesorectal Excision and 3‐Year Disease‐Free Survival in Rectal Cancer: The TaLaR Randomized Clinical Trial,” Journal of the American Medical Association 333, no. 9 (2025): 774–783. 10.1001/jama.2024.24276 PMC11880948 39847361

[27] C. K. Liao , Y. L. Yu , Y. T. Kuo , et al., “Transanal Versus Transabdominal Total Mesorectal Excision for Rectal Cancer in Minimally Invasive Surgery: Meta‐Analysis,” BJS Open 9, no. 6 (2025): zraf111. 10.1093/bjsopen/zraf111 PMC12641135 41277267

[28] H. H. Wasmuth , A. E. Faerden , T. A. Myklebust , et al., “Transanal Total Mesorectal Excision for Rectal Cancer Has Been Suspended in Norway,” British Journal of Surgery 107, no. 1 (2020): 121–130. 10.1002/bjs.11459 31802481

[29] S. E. van Oostendorp , H. J. E. Belgers , B. T. Bootsma , et al., “Locoregional Recurrences After Transanal Total Mesorectal Excision of Rectal Cancer During Implementation,” British Journal of Surgery 107, no. 9 (2020): 1211–1220. 10.1002/bjs.11525 PMC7496604 32246472

[30] S. E. van Oostendorp , H. J. E. Belgers , J. C. Hol , et al., “The Learning Curve of Transanal Total Mesorectal Excision for Rectal Cancer Is Associated With Local Recurrence: Results From a Multicentre External Audit,” Colorectal Disease 23, no. 8 (2021): 2020–2029. 10.1111/codi.15722 PMC8453958 33969621

[31] T. Matsuda , I. Takemasa , H. Endo , et al., “Local Recurrence of Rectal Cancer After Transanal Total Mesorectal Excision and Risk Factors: A Nationwide Multicenter Cohort Study in Japan,” Annals of Surgery Open 5, no. 1 (2024): e369. 10.1097/AS9.0000000000000369 PMC11175902 38883940

[32] S. Yamauchi , A. Shiomi , C. Matsuda , et al., “Robotic‐Assisted Colectomy for Right‐Sided Colon Cancer: Short‐Term Surgical Outcomes of a Multi‐Institutional Prospective Cohort Study in Japan,” Annals of Gastroenterological Surgery 7, no. 6 (2023): 932–939. 10.1002/ags3.12694 PMC10623957 37927933

[33] M. Numata , J. Watanabe , A. Ishibe , et al., “Surgical Outcomes of a Prospective, Phase 2 Trial of Robotic Surgery for Resectable Right‐Sided Colon Cancer (The ROBOCOLO Trial),” Annals of Gastroenterological Surgery 8, no. 1 (2024): 80–87. 10.1002/ags3.12718 PMC10797943 38250687

[34] K. Morohara , H. Katsuno , T. Endo , et al., “Short‐Term Surgical Outcomes of Robot‐Assisted Colectomy for Colon Cancer Using the Hinotori Surgical Robot System,” Annals of Coloproctology 41, no. 1 (2025): 97–103. 10.3393/ac.2024.00871.0124 PMC11897604 40044114

